# Pancreatic Cancer Surveillance and Survival of High-Risk Individuals

**DOI:** 10.1001/jamaoncol.2024.1930

**Published:** 2024-07-03

**Authors:** Amanda L. Blackford, Marcia Irene Canto, Mohamad Dbouk, Ralph H. Hruban, Bryson W. Katona, Amitabh Chak, Randall E. Brand, Sapna Syngal, James Farrell, Fay Kastrinos, Elena M. Stoffel, Anil Rustgi, Alison P. Klein, Ihab Kamel, Elliot K. Fishman, Jin He, Richard Burkhart, Eun Ji Shin, Anne Marie Lennon, Michael Goggins

**Affiliations:** 1 Department of Oncology, The Sol Goldman Pancreatic Cancer Research Center, Johns Hopkins Medical Institutions, Baltimore, Maryland; 2 Department of Pathology, The Sol Goldman Pancreatic Cancer Research Center, Johns Hopkins Medical Institutions, Baltimore, Maryland; 3 Department of Medicine (Gastroenterology), The Sol Goldman Pancreatic Cancer Research Center, Johns Hopkins Medical Institutions, Baltimore, Maryland; 4 Department of Radiology, The Sol Goldman Pancreatic Cancer Research Center, Johns Hopkins Medical Institutions, Baltimore, Maryland; 5 Department of Surgery, The Sol Goldman Pancreatic Cancer Research Center, Johns Hopkins Medical Institutions, Baltimore, Maryland; 6 Division of Gastroenterology, Department of Medicine, Abramson Cancer Center, University of Pennsylvania Perelman School of Medicine, Philadelphia; 7 Division of Gastroenterology and Liver Disease, University Hospitals Cleveland Medical Center, Case Western Reserve University, Cleveland, Ohio; 8 Division of Gastroenterology, Hepatology and Nutrition, University of Pittsburgh Medical Center, Pennsylvania; 9 Cancer Genetics and Prevention, Medical Oncology, Dana-Farber Cancer Institute, Boston, Massachusetts; 10 Division of Gastroenterology, Brigham and Women’s Hospital and Harvard Medical School, Boston, Massachusetts; 11 Yale Center for Pancreatic Disease, Section of Digestive Disease, Yale University, New Haven, Connecticut; 12 Division of Digestive and Liver Diseases, Herbert Irving Comprehensive Cancer Center, Vagelos College of Physicians and Surgeons, Columbia University Irving Medical Center, New York, New York; 13 Division of Gastroenterology and Hepatology, Department of Internal Medicine, University of Michigan, Ann Arbor

## Abstract

**Importance:**

Pancreatic ductal adenocarcinoma (PDAC) is a deadly disease with increasing incidence. The majority of PDACs are incurable at presentation, but population-based screening is not recommended. Surveillance of high-risk individuals for PDAC may lead to early detection, but the survival benefit is unproven.

**Objective:**

To compare the survival of patients with surveillance-detected PDAC with US national data.

**Design, Setting, and Participants:**

This comparative cohort study was conducted in multiple US academic medical centers participating in the Cancer of the Pancreas Screening program, which screens high-risk individuals with a familial or genetic predisposition for PDAC. The comparison cohort comprised patients with PDAC matched for age, sex, and year of diagnosis from the Surveillance, Epidemiology, and End Results (SEER) program. The Cancer of the Pancreas Screening program originated in 1998, and data collection was done through 2021. The data analysis was performed from April 29, 2022, through April 10, 2023.

**Exposures:**

Endoscopic ultrasonography or magnetic resonance imaging performed annually and standard-of-care surgical and/or oncologic treatment.

**Main Outcomes and Measures:**

Stage of PDAC at diagnosis, overall survival (OS), and PDAC mortality were compared using descriptive statistics and conditional logistic regression, Cox proportional hazards regression, and competing risk regression models. Sensitivity analyses and adjustment for lead-time bias were also conducted.

**Results:**

A total of 26 high-risk individuals (mean [SD] age at diagnosis, 65.8 [9.5] years; 15 female [57.7%]) with PDAC were compared with 1504 SEER control patients with PDAC (mean [SD] age at diagnosis, 66.8 [7.9] years; 771 female [51.3%]). The median primary tumor diameter of the 26 high-risk individuals was smaller than in the control patients (2.5 [range, 0.6-5.0] vs 3.6 [range, 0.2-8.0] cm, respectively; *P* < .001). The high-risk individuals were more likely to be diagnosed with a lower stage (stage I, 10 [38.5%]; stage II, 8 [30.8%]) than matched control patients (stage I, 155 [10.3%]; stage II, 377 [25.1%]; *P* < .001). The PDAC mortality rate at 5 years was lower for high-risk individuals than control patients (43% vs 86%; hazard ratio, 3.58; 95% CI, 2.01-6.39; *P* < .001), and high-risk individuals lived longer than matched control patients (median OS, 61.7 [range, 1.9-147.3] vs 8.0 [range, 1.0-131.0] months; 5-year OS rate, 50% [95% CI, 32%-80%] vs 9% [95% CI, 7%-11%]).

**Conclusions and Relevance:**

These findings suggest that surveillance of high-risk individuals may lead to detection of smaller, lower-stage PDACs and improved survival.

## Key Points

**Question:**

What is the association of selective pancreatic cancer surveillance with survival?

**Findings:**

In this cohort study comparing 26 individuals with a genetic or familial predisposition undergoing pancreatic cancer surveillance and 1504 matched control patients, surveillance led to a significantly higher proportion of stage I cancers (31% vs 10%), longer 5-year survival rate (50% vs 9%), and lower pancreatic cancer–specific mortality rate (43% vs 86%).

**Meaning:**

These findings suggest that selective surveillance of individuals at high risk for pancreatic cancer may improve clinical outcomes.

## Teaser

This cohort study compares survival of high-risk patients with screen-detected pancreatic cancer with a matched cohort from the Surveillance, Epidemiology, and End Results program.

## Introduction

Pancreatic ductal adenocarcinoma (PDAC) is a highly lethal disease.^1^ In the US, it is the eighth most common cancer but ranks third as a cause of cancer death.^2^ While the overall survival (OS) for individuals with PDAC has improved in recent years, it remains poor, with approximately 11% alive 5 years after diagnosis.^2,3^ The majority of PDACs are diagnosed at an advanced, often locally unresectable or metastatic stage, which in turn directly affects survival. However, population-based screening for PDAC in average-risk, asymptomatic individuals is not recommended.^4^

For the past 2 decades, there has been increasing interest and accumulating data on the diagnostic yield of selective surveillance in high-risk individuals with a family history of PDAC or with a genetic predisposition due to an inherited pathogenic variant.^5,6,7,8,9^ The Cancer of the Pancreas Screening (CAPS) program, consisting of high-risk individuals with inherited genetic variants or familial pancreatic cancer kindreds, reported 10 PDACs diagnosed in 1461 study participants, with 78% diagnosed during surveillance at stage I using endoscopic ultrasonography (EUS) and/or magnetic resonance imaging (MRI).^10^ The 20-year Dutch program reported its experience with 347 carriers of deleterious germline *CDKN2A* variants who underwent surveillance primarily with MRI. Among study participants who developed PDAC, 33.3% were diagnosed with stage I disease, and 83% had resectable disease.^11^

Although these data suggest that surveillance can detect earlier disease, the effectiveness of surveillance for improvement of PDAC survival in high-risk individuals is unproven.^12^ We aimed to determine whether there was a shift to lower-stage PDAC and an associated improved long-term survival in high-risk individuals compared with matched control patients after considering factors known to be associated with survival.

## Methods

### Study Population

This comparative cohort study examined high-risk individuals enrolled in the CAPS program,^13^ an ongoing, multicenter, prospective, institutional review board–approved cohort study. CAPS originated in 1998 as a single-center clinical program and observational study at the Johns Hopkins Hospital.^14^ CAPS1 was a pilot study involving 38 high-risk individuals^14^; CAPS2, a prospective cohort study of 78 high-risk individuals and 149 control participants^15^; and CAPS3 (2007-2010), a multicenter study that compared the diagnostic yield of computed tomography (CT), MRI, and EUS in 225 high-risk individuals. CAPS4 (2008-2013) was a Johns Hopkins single-center study that evaluated the yield of pancreatic surveillance and biomarkers for early detection. The ongoing CAPS5 (since 2014) is a multicenter study of 8 academic centers.^5,10^ Experts performed EUS and MRI at each site. The CAPS imaging protocols and reporting have been standardized since CAPS3 and have followed a 2004 CAPS consensus of a joint National Cancer Institute and Specialized Program of Research Excellence–sponsored workshop. The CAPS cohort studies were approved by the Johns Hopkins institutional review board. This study was exempt from review because it used prospectively collected data from the prior CAPS studies that obtained patients’ informed consent. High-risk individuals eligible for surveillance provided written informed consent. This study followed the Strengthening the Reporting of Observational Studies in Epidemiology (STROBE) reporting guideline.

#### High-Risk Individuals

High-risk individuals were eligible for surveillance if they were asymptomatic at baseline. Participants underwent baseline evaluation with a comprehensive questionnaire, EUS, and abdominal contrast-enhanced CT or 1.5T or 3T contrast-enhanced MRI with magnetic resonance cholangiopancreatography according to study protocol (1998-2013) or international CAPS Consortium consensus guidelines (since 2013).^16,17^ After baseline, participants continued annual surveillance unless there was a clinical need for a shortened interval follow-up based on detected abnormalities.

The high-risk individuals were enrolled based on either family history criteria, which included individuals who had at least 1 first-degree relative with PDAC and who were part of a kindred with at least 1 pair of affected first-degree relatives (familial pancreatic cancer kindred), or germline gene status criteria (carriers of a germline deleterious variant in a gene associated with hereditary pancreatic cancer [*ATM*, *BRCA1*, *BRCA2*, *CDKN2A*, *PALB2*, or *STK11*]). A total of 1731 high-risk individuals were enrolled in the CAPS studies between 1998 and June 2021.^5,10,14,15,18^ The current study includes the 26 high-risk individuals diagnosed with PDAC during this period (hereafter, high-risk individuals with PDAC) (eFigure 1, eTable 1 in Supplement 1). A more detailed description of this study cohort is available in Dbouk et al.^19^

When an high-risk individual developed a suspected pancreatic neoplasm (solid mass, cyst with worrisome features, or lesion with suspicious or positive cytology), surgical treatment was performed after multidisciplinary discussion and shared decision-making with the participant. Surgery was performed by expert pancreaticobiliary surgeons, and final diagnoses were made by an expert pathologist at each site.

#### Matched Control Patients With PDAC

The high-risk individuals with PDAC were matched to control patients with PDAC selected from the Surveillance, Epidemiology, and End Results (SEER) 18 registry grouping (November 2020 submission). The SEER data are considered nonhuman participant research. Data are anonymized, and no patient contact is involved in their collection.

We used the MatchIt package in R, version 4.2.3 (R Project for Statistical Computing) to select SEER control patients with PDAC who matched exactly on age, sex, and year of diagnosis to high-risk individuals with PDAC. All the high-risk individuals with PDAC were White, so control patients were matched on White race. Participants self-reported race and ethnicity information on the baseline survey. The matching approach identified all unique combinations of covariates to form subclasses, and only subclasses with both high-risk individuals with PDAC and SEER control patients with PDAC were included in the final cohort.

### Statistical Analysis

Data were collected from 1998 through 2021. The data analysis was performed from April 29, 2022, through April 10, 2023. The primary analyses were comparisons of American Joint Committee on Cancer PDAC tumor stage at diagnosis (using the 6th, 7th, and 8th editions of the *American Joint Committee on Cancer Staging Manual*, depending on year of diagnosis), OS, and PDAC mortality between high-risk individuals with PDAC and SEER control patients. Clinical and demographic characteristics, including stage at diagnosis, were summarized using descriptive statistics and tested for differences between cohorts using conditional logistic regression models with matching weights and clustered on the covariate subclasses. Overall survival was calculated as the time from diagnosis to last follow-up or death and estimated using the Kaplan-Meier method. The OS was compared between groups using a Cox proportional hazards regression model that included model weights and a cluster-robust standard error estimation accounting for the matching. Mortality specifically from PDAC was compared between high-risk individuals with PDAC and SEER control patients using competing-risks regression for clustered data, with death from other causes considered a competing event (package crrSC in R, version 4.2.3). A 2-sided *P* < .05 was considered significant for all analyses.

#### Sensitivity Analyses

Three sets of sensitivity analyses for the primary analyses were conducted after sequentially excluding specific subgroups of high-risk individuals with PDAC (eFigure 1 in Supplement 1). These analyses included (1) rematching on primary tumor location after excluding 2 high-risk individuals with PDAC who had dropped out of surveillance and presented at an outside institution with metastatic disease but for whom primary tumor location within the pancreas was unknown, (2) excluding 4 more high-risk individuals who presented with PDAC after having dropped out of surveillance (over approximately 3 months beyond the recommended annual surveillance), and (3) limiting analyses to the subgroup of 18 high-risk individuals with screen-detected PDAC without metastatic disease who underwent pancreatic resection.

#### Adjustment for Lead-Time Bias

To adjust for potential lead-time bias in the high-risk individuals with PDAC, we included an additional sensitivity analysis using the method described in Duffy et al.^20^ We considered mean sojourn times of 3, 6, and 12 months and reestimated survival probabilities and hazard ratios (HRs) using the adjusted survival times. Further details about the SEER cohort and the sensitivity analysis are provided in the eMethods in Supplement 1.

## Results

### Baseline Characteristics

The characteristics of 26 high-risk individuals with PDAC (mean [SD] age at diagnosis, 65.8 [9.5] years; 15 female [57.7%] and 11 male [42.3%]; all White race) are detailed in Table 1. Twenty of the 26 (76.9%) high-risk individuals with PDAC were diagnosed after a median of 5.8 years (range, 1.1-11.2 years) from study entry, while 6 (23.1%) were diagnosed at baseline (Table 1). Nearly one-half (12 of 26 [46.2%]) of the PDACs in high-risk individuals were located in the pancreatic head (Table 2). Of the high-risk individuals with PDAC, 3 (11.5%) underwent neoadjuvant therapy. Patient and tumor characteristics of both the matched cohort of 1504 White patients (mean [SD] age at diagnosis, 66.8 [7.9] years; 771 female [51.3%] and 733 male [48.7%]) and entire eligible SEER PDAC cohort of 66 987 individuals (mean [SD] age at diagnosis, 67.9 [11.3] years; 32 242 female [48.1%] and 34 745 male [51.9%]) are included for comparison (Table 2). There were small differences in the distribution of the primary tumor within the pancreas and, thus, the type of surgery performed compared with matched SEER control patients (Table 2). The high-risk individuals with PDAC had fewer well-differentiated cancers compared with matched SEER control patients (1 of 19 [5.3%] vs 74 of 509 [14.5%]; *P* = .03). Median tumor size was significantly smaller in high-risk individuals with PDAC compared with matched SEER control patients (2.5 [range, 0.6-5.0] vs 3.6 [range, 0.2-8.0] cm, respectively; *P* < .001).

**Table 1.** Characteristics of 26 High-Risk Individuals Diagnosed With PDAC During Screening, 1998-2021

Characteristic	No. of high-risk individuals with PDAC (%)
Age at diagnosis, mean (SD), y	65.8 (9.5)
Sex	
Female	15 (57.7)
Male	11 (42.3)
White race	26 (100)
Familial pancreatic cancer	16 (61.5)
Germline variant carriers	10 (38.5)
Follow-up, median (range), y	2.5 (0.2-13.0)
Time from baseline to diagnosis for nonprevalent cases, median (range), y^a^	5.8 (1.1-11.2)
PDAC diagnosed at baseline, prevalent cases	6 (23.1)

Abbreviation: PDAC, pancreatic ductal adenocarcinoma.

^a^ Number of nonprevalent cases was 20.

**Table 2.** Characteristics of High-Risk Individuals With PDAC, Matched SEER Control Patients With PDAC, and the Full SEER Cohort Eligible for Matching

Characteristic	No. (%)			*P* value^a^
	High-risk individuals (n = 26)	Matched SEER control patients with PDAC (n = 1504)	Eligible SEER cohort (n = 66 987)	
Age at diagnosis, mean (SD), y	65.8 (9.5)	66.8 (7.9)	67.9 (11.3)	NA
Sex				
Female	15 (57.7)	771 (51.3)	32 242 (48.1)	NA
Male	11 (42.3)	733 (48.7)	34 745 (51.9)	
Year of diagnosis				
1998-2010	6 (23.1)	325 (21.6)	23 929 (35.7)	NA
2011-2015	7 (26.9)	378 (25.1)	22 985 (34.3)	
2016-2021^b^	13 (50.0)	801 (53.3)	20 073 (30.0)	
Tumor location				
Head	12 (50.0)	767 (51.0)	35 117 (52.4)	.002
Body or tail	12 (50.0)	448 (29.8)	18 691 (27.9)	
Other	0	289 (19.2)	13 179 (19.7)	
Missing	2	0	0	
Type of surgery				
Whipple	9 (50.0)	243 (63.0)	9713 (60.9)	.03
Distal	8 (44.4)	108 (28.0)	4493 (28.2)	
Total pancreatectomy	1 (5.6)	35 (9.1)	1754 (11.0)	
Missing^c^	8	1118	51 027	
Tumor grade				
1 (Well differentiated)	1 (5.3)	74 (14.5)	4012 (17.0)	.03
2 (Moderately differentiated)	15 (78.9)	247 (48.5)	9941 (42.0)	
3 or 4 (Poorly differentiated, undifferentiated, or anaplastic)	3 (15.8)	188 (36.9)	9699 (41.0)	
Missing	7	995	43 335	
Tumor size, median (range), cm	2.5 (0.6-5.0)	3.6 (0.2-8.0)	3.6 (0.0-8.0)^d^	<.001
T stage				
1	6 (27.3)	57 (4.4)	2977 (5.2)	<.001
2	10 (45.5)	425 (32.8)	16 563 (29.0)	
3	5 (22.7)	530 (40.9)	25 144 (44.0)	
4	1 (4.5)	283 (21.9)	12 484 (21.8)	
Missing	4	209	9819	
N stage				
0	12 (57.1)	771 (61.9)	35 328 (61.5)	.56
1	9 (42.9)	446 (35.8)	21 566 (37.5)	
2	0	29 (2.3)	590 (1.0)	
Missing	5	258	9503	
No. of positive nodes per total nodes examined, mean (SD)	0.06 (0.10)	0.15 (0.19)	0.15 (0.20)	.004
M stage				
0	19 (73.1)	695 (46.2)	32 665 (48.8)	.01
1	7 (26.9)	809 (53.8)	34 322 (51.2)	
AJCC stage				
I	10 (38.5)	155 (10.3)	7508 (11.2)	<.001
II	8 (30.8)	377 (25.1)	18 056 (27.0)	
III	1 (3.8)	163 (10.8)	7101 (10.6)	
IV	7 (26.9)	809 (53.8)	34 322 (51.2)	
AJCC stage				
Ia	6 (23.1)	37 (2.5)	2187 (3.3)	<.001
Ib	4 (15.4)	118 (7.8)	5321 (7.9)	
IIa	2 (7.7)	154 (10.2)	7622 (11.4)	
IIb	6 (23.1)	223 (14.8)	10 434 (15.6)	
III	1 (3.8)	163 (10.8)	7101 (10.6)	
IV	7 (26.9)	809 (53.8)	34 322 (51.2)	
Summary stage				
Localized	12 (46.2)	309 (20.5)	15 130 (22.6)	.01
Regional	7 (26.9)	386 (25.7)	17 535 (26.2)	
Distant	7 (26.9)	809 (53.8)	34 322 (51.2)	
Received neoadjuvant chemotherapy				
Yes	3 (11.5)	985 (65.5)	39 998 (59.7)	<.001
No, unknown, no surgery performed	23 (88.5)	519 (34.5)	26 989 (40.3)	

Abbreviations: AJCC, American Joint Committee on Cancer; NA, not applicable; PDAC, pancreatic ductal adenocarcinoma; SEER, Surveillance, Epidemiology, and End Results.

^a^ *P* values for differences between high-risk individuals with PDAC and matched SEER control patients with PDAC estimated from conditional logistic regression models. *P* values were not computed for age, sex, and year of diagnosis, as they were the variables included in the matching algorithm.

^b^ SEER data were available up through 2019. Each high-risk individual with PDAC diagnosed in 2020 or 2021 was matched to a patient in SEER diagnosed in 2019.

^c^ The 8 high-risk individuals with PDAC did not undergo surgery. In the SEER cohorts, either patients did not undergo surgery or information about surgery was not available.

^d^ Tumor sizes of 0 indicate that no mass or tumor was found, for example, when a tumor of a stated primary site is not found, but the tumor has metastasized.

### Stage Shift Analysis

**Figure 1.**
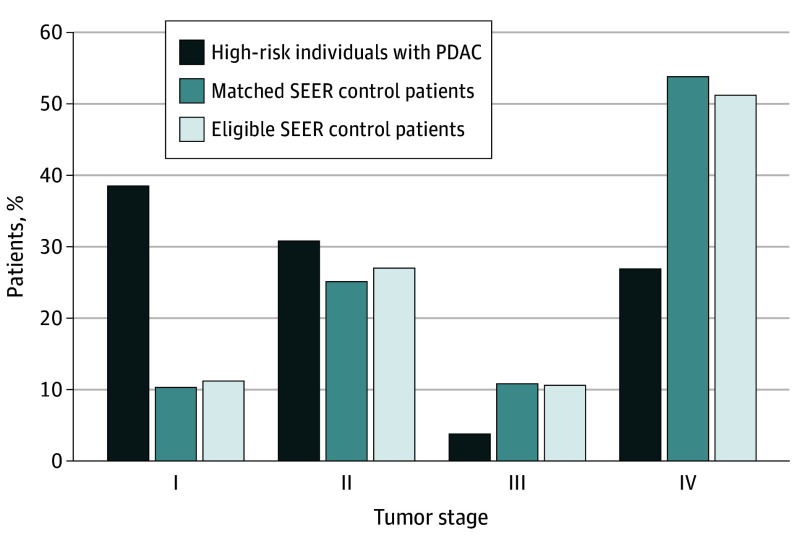
Tumor Stage at Diagnosis Frequency distribution of tumor stage at diagnosis for 26 high-risk individuals with pancreatic ductal adenocarcinoma (PDAC); 1504 matched Surveillance, Epidemiology, and End Results (SEER) control patients with PDAC; and the pool of 66 987 eligible SEER patients with PDAC from which the matched case patients were drawn. *P* < .001 for high-risk individuals with PDAC vs matched SEER control patients.

There was a significant shift in the American Joint Committee on Cancer tumor stage of high-risk individuals with PDAC toward lower disease stages compared with the matched SEER control patients (stage I, 10 of 26 [38.5%] vs 155 of 1504 [10.3%]; stage II, 8 of 26 [30.8%] vs 377 of 1504 [25.1%]; *P* < .001) (Figure 1). Similarly, T stage for high-risk individuals with PDAC was lower compared with matched SEER control patients, with 6 of 22 (27.3%) vs 57 of 1295 (4.4%) with T1 stage, respectively (Table 2). Furthermore, fewer high-risk individuals with PDAC had distant metastases at diagnosis (M1 stage) compared with matched SEER control patients (7 of 26 [26.9%] vs 809 of 1504 [53.8%]; *P* = .01).

### Survival Comparison, Sensitivity Analyses, and Adjustment for Lead-Time Bias

**Figure 2.**
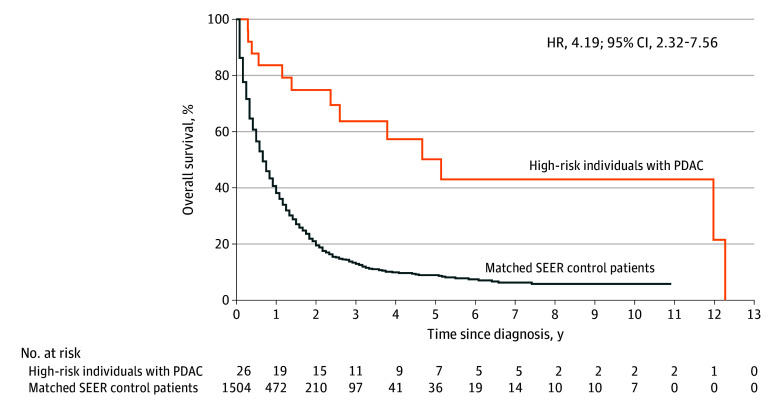
Overall Survival for Pancreatic Ductal Adenocarcinoma (PDAC) in High-Risk Individuals and Matched Surveillance, Epidemiology, and End Results (SEER) Control Patients HR indicates hazard ratio.

Overall survival was higher in the 26 high-risk individuals with PDAC (median, 61.7 months; range, 1.9-147.3 months) compared with matched SEER controls (median, 8.0 months; range, 1.0-131.0 months; HR, 4.19; 95% CI, 2.32-7.56; *P* < .001) (Figure 2). One- and 5-year survival probabilities were 84% (95% CI, 70%-100%) and 50% (95% CI, 32%-80%), respectively, for high-risk individuals with PDAC and 38% (95% CI, 36%-41%) and 9% (95% CI, 7%-11%), respectively, for SEER control patients (Table 3). Moreover, median OS in the 20 high-risk individuals whose PDAC was screen detected was 144 months (range, 3.6-147.3 months), while matched SEER control patients had a median OS of 9 months (range, 1.0-123.0 months). One- and 5-year survival probabilities were 95% (95% CI, 86%-100%) and 61% (95% CI, 40%-93%), respectively, in the high-risk individuals with screen-detected PDAC and 41% (95% CI, 36%-46%) and 9% (95% CI, 5%-14%), respectively, in matched SEER control patients (eTable 3 in Supplement 1). Similar results were observed when matching was limited to the high-risk individuals with PDAC with known primary tumor location (eTable 1 in Supplement 1) and for the 18 surgically treated high-risk individuals with screen-detected PDAC (eTable 2, eFigure 2 in Supplement 1). Additional survival analysis and results are described in the eMethods in Supplement 1.

**Table 3.** Comparison of OS in High-Risk Individuals With PDAC and Matched SEER Control Patients With Additional Sensitivity Analysis Accounting for Potential Lead-Time Bias

Group	OS, median (range), mo	Survival probability (range), %		Observed		Lead-time bias					
						3 mo		6 mo		12 mo	
		1 y	5 y	HR (95% CI)^a^	*P* value	HR (95% CI)^a^	*P* value	HR (95% CI)^a^	*P* value	HR (95% CI)^a^	*P* value
High-risk individuals with PDAC (n = 26)	61.7 (1.9-147.3)	84 (70-100)	50 (32-80)	1 [Reference]	<.001	1 [Reference]	<.001	1 [Reference]	<.001	1 [Reference]	<.001
Matched SEER control patients with PDAC (n = 1504)	8.0 (1.0-131.0)	38 (36-41)	9 (7-11)	4.19 (2.32-7.56)		3.91 (2.12-7.21)		3.69 (1.97-6.91)		3.34 (1.74-6.40)	

Abbreviations: HR, hazard ratio; OS, overall survival; PDAC, pancreatic ductal adenocarcinoma; SEER, Surveillance, Epidemiology, and End Results.

^a^ The HRs from Cox proportional hazards models were estimated using cluster-robust standard errors clustered on the matched subclass and including subclass-specific weights. The high-risk individuals with PDAC were matched to SEER control patients on age, sex, and year of diagnosis.

### Tumor-Specific Mortality

The cumulative probability of death from PDAC at 1 and 5 years was 16% and 43% for high-risk individuals with PDAC and 59% and 86% for matched SEER control patients, respectively (HR, 3.58; 95% CI, 2.01-6.39; *P* < .001). For the surgically treated high-risk individuals with screen-detected PDAC, the corresponding cumulative probabilities were 6% and 29% at 1 and 5 years, respectively.

## Discussion

Incidence rates for PDAC over the past decade have continued to increase, with minimal improvement in survival worldwide despite advances in health care.^1,4^ Unlike common cancers, such as breast, colorectal, and lung cancer, screening for early detection and prevention of pancreatic cancer remains a challenge because it is a relatively rare disease that can be difficult to diagnose, even when symptomatic.^12^ The overall and cancer-specific 5-year survival for patients with PDAC are 11% and 13%, respectively.^21^ However, patients with resectable stage Ia PDAC have a 5-year survival rate of approximately 84%,^22^ which underscores the potential for improved outcomes through early detection.

Screening of average-risk individuals for PDAC is not recommended by the US Preventive Services Task Force.^4^ Because PDAC is a rare, albeit deadly disease, a randomized clinical trial comparing surveillance vs no surveillance in high-risk individuals is not feasible. Selective surveillance of high-risk individuals has been 1 proposed alternative to population-based screening in the past 2 decades. Using EUS, MRI, and/or CT, 25 surveillance studies involving more than 3000 high-risk individuals showed that early detection of asymptomatic PDAC is feasible.^23^ In a 2019 clinical practice update, the American Gastroenterological Association endorsed selective surveillance in high-risk individuals.^24^ In a 2022 clinical guideline, the American Society for Gastrointestinal Endoscopy recommended selective surveillance of high-risk individuals based on scientific review and a Grading of Recommendations Assessment, Development, and Evaluation methodology.^23,25^

The findings of our cohort study show that high-risk individuals who underwent annual or semiannual surveillance EUS and MRI and who were diagnosed with PDAC had a greater likelihood of having smaller, lower-stage tumors; lower PDAC-specific mortality; and better OS (5-year rate, 50%) compared with a national cohort (5-year rate, 9%) matched for age, sex, race, and year of diagnosis and with similar tumor location and type of surgery. The adjusted HRs showed a 4-fold higher chance of being alive for screened high-risk individuals.

One study reported improved outcomes for surveillance-detected PDAC in a cohort with the Dutch *CDKN2A/p16* founder variant compared with matched patients with PDAC from the Dutch National Cancer Registry after adjusting for lead-time bias.^26^ In this Dutch study, surveillance led to a shift to a lower stage (38.7% stage I) and higher 5-year survival rate (32.4%). Similarly, in the current study, we also found that the improved effects of early detection and treatment of PDAC in high-risk individuals undergoing surveillance may be due to lead-time bias since disease-specific mortality was also decreased and sensitivity analyses showed similar findings. We also report a reduction in PDAC mortality that supports selective surveillance for a rare but deadly cancer, which compares favorably with screening for more common cancers arising from the breast^27^ or colon.^28,29,30^

Survival after a PDAC diagnosis among high-risk individuals undergoing surveillance in the CAPS program is higher than reported in other established programs.^7,11,26^ Although PDAC surveillance programs^7,10,11^ have shown a greater proportion of stage I cancers (33%-40% of cases) and improved resectability (60%-83%) for all patients with screen-detected PDAC compared with patients with PDAC diagnosed in the general population or outside of surveillance, the reported survival benefit is highly variable, with median survival ranging from 18 months in the other studies to 61.7 months in our study.

What accounts for these differences in survival among high-risk surveillance cohorts is an important unanswered question. One of the main differences between the 2 cohorts is the number of stage I cases, which may be the result of differences in the surveillance method used or in disease biology. Regarding surveillance tests, EUS is known to have a higher resolution compared with MRI and CT and better accuracy for detecting small solid pancreatic cancers than MRI, although the available data are limited.^5,31,32^ The Dutch surveillance program that reported PDAC outcomes of individuals with deleterious germline *CDKN2A* variants primarily relied on MRI for surveillance, with optional EUS.^11,33^ Seventeen percent of incident PDACs in the Dutch cohort presented as interval cancers.^11^ Furthermore, a retrospective review of imaging abnormalities in 28 of 2552 high-risk individuals who developed PDAC while participating in 1 of 16 surveillance programs from 7 countries (Australia, Germany, Israel, Italy, the Netherlands, Spain, and the US) found that almost one-half of the high-grade dysplasia or PDAC lesions were detected before the next scheduled surveillance visit, at a median of approximately 11 months after an unremarkable imaging examination.^32^ Differences between surveillance programs^32^ with respect to how the imaging tests are used (magnetic resonance/magnetic resonance cholangiopancreatography, EUS, or both at each visit); surveillance intervals (6 or 12 months); and other aspects of their radiologic, surgical, and oncologic care may have contributed to the variability in outcomes. Interval cancers detected in surveillance programs of high-risk individuals highlight the continuing need to develop more sensitive diagnostic tools and standardize or modify surveillance methods.

Another potential reason for variability in the survival benefit from surveillance of high-risk individuals is variability in how patients with pancreatic cancer are treated. For example, some patients in the Dutch cohort with resectable PDAC declined surgery, and the more recently diagnosed high-risk individuals in our cohort were more likely to have undergone neoadjuvant therapy (11.5% in our cohort vs 0% in the Rotterdam cohort^32^ and 11% in the *CDKN2A* cohort). Targeted chemotherapy with poly-ADP ribose polymerase inhibitors and platinum-based agents for high-risk individuals with germline *BRCA*/*PALB2* variants can potentially improve progression-free survival.^34,35^ Finally, differences in tumor biology may contribute to the wide range of reported PDAC survival. Germline gene status may affect the timeline from preclinical invasive cancer, to a detectable localized tumor, to metastatic or locally advanced PDAC.

While the current study provides encouraging results and underscores the potential of surveillance and early detection to improve survival of patients with PDAC in a well-defined high-risk cohort, it is unclear whether surveillance outside specialized centers can be recommended. The benefits of surveillance have to be weighed against potential harms. Although morbidity of pancreatic surgery may be substantial, morbidity after pancreatic resection of surveillance-detected lesions in high-risk individuals in high-volume surgical centers (19.9%)^25^ has been lower or comparable to known morbidity rates after PDAC resection (30%-60%),^36^ with no operative mortality to date.^25,37^ However, screening for a low-prevalence disease may lead to false-positive results. The diagnostic yield (finding PDAC or high-grade dysplasia at resection) of surveillance-detected lesions is low (29% of those undergoing surgical resection,^38^ or 8.6% of high-risk individuals^23^), despite expert multidisciplinary recommendation and shared decision-making. False-negative test results are also still a problem, and radiologists frequently (50%-62%) miss pancreatic cancers presenting with atypical or subtle signs on CT or MRI,^39,40,41^ which may lead to delayed diagnosis and advanced disease. In a retrospective review of prior negative MRI findings for carriers of germline *CDKN2A* pathogenic variants who developed PDAC, mild pancreatic ductal dilatation was an unrecognized subtle abnormality.^42^ Nonetheless, nearly one-half of high-risk individuals who developed neoplastic progression while under surveillance had no prior pancreatic lesions detected by imaging studies.^7^ In addition, the psychological burden of annual surveillance of high-risk individuals should be considered, but it appears to be low,^43^ and surveillance may be associated with decreased cancer worry after initiating it. Finally, the cost of selective surveillance of high-risk individuals with EUS and MRI are high, and access to surveillance programs is currently limited. Four studies consistently found that surveillance of high-risk individuals was cost-effective compared with no surveillance,^44,45,46,47^ but there is substantial variability in cost.^23^

A multimodal approach to PDAC surveillance that estimates individualized risk using patient demographics, clinical tests, imaging, genomics,^48,49^ biomarkers, and artificial intelligence is an as-yet unrealized “holy grail” for achieving better outcomes with the highest benefit and least cost and harm. Serum biomarkers using proteins or circulating tumor DNA have the potential to improve surveillance.^50,51,52,53,54,55,56,57^ To improve on current pancreas surveillance with EUS and MRI, these biomarkers must be low cost, highly specific, and sufficiently sensitive to detect early-stage PDAC. In the future, these noninvasive tests might be incorporated into a surveillance strategy for high-risk individuals between or at the time of scheduled MRI and/or EUS visits or widely applied for screening average-risk individuals. Furthermore, artificial intelligence is gaining momentum in improving the sensitivity and specificity of surveillance CT, MRI, and EUS.^58,59^ Finally, artificial intelligence can also help to estimate PDAC risk through electronic health records,^60^ refine the identification of high-risk individuals, and potentially enable a broader targeted surveillance program in much larger populations for wider implementation.

### Strengths and Limitations

Our study has several strengths, including multiple centers, a well-defined cohort of high-risk individuals and control patients, standardized surveillance methods combining EUS and MRI, a long-standing clinical and research surveillance program with multidisciplinary expert teams, and analytic methods that address the potential effects of bias. Our study also has several limitations, including enrollment at academic referral centers and limited generalizability, limited racial and ethnic diversity, a relatively small number of high-risk individuals who progressed to PDAC, and lack of a control arm of unscreened high-risk individuals. The National Cancer Institute SEER database provides a wealth of national cancer data, but it has limitations that include incomplete neoadjuvant and adjuvant therapy data and variations in data reporting, among others. Furthermore, it is possible that patients who participate in pancreas surveillance may be otherwise healthier (with respect to diet, exercise, and smoking) and so may have better clinical outcomes than those in the SEER database.

## Conclusions

In this comparative cohort study, surveillance of high-risk individuals for PDAC using EUS and MRI within established programs at academic centers was observed to lead to the detection of smaller pancreatic cancers, a greater number of patients with stage I disease, lower mortality, and a much higher likelihood of long-term survival than unscreened patients in the general population diagnosed with PDAC. These findings suggest that selective surveillance of individuals at high risk for pancreatic cancer may improve clinical outcomes.

## Notes

Supplement 1.
eMethods
**eTable 1.** Characteristics of 24 HRIs With PDAC With Known Tumor Location (Left) and Those Diagnosed During Screening (Right) Compared With Matched SEER Control Patients **eTable 2.** Comparison of 18 HRIs With Surgically Treated Surveillance-Detected PDAC and Matched SEER Control Patients With Surgically Treated PDAC **eTable 3.** Comparison of Overall PDAC Survival Among Subsets of HRIs With PDAC With Matched SEER Control Patients, With Additional Sensitivity Analysis Accounting for Potential Lead-Time Bias **eFigure 1.** Study Flow Chart **eFigure 2.** Overall Survival of 18 Surveillance-Detected HRIs With PDAC and Matched SEER Control Patients With Nonmetastatic, Resectable Disease

Supplement 2.
Data Sharing Statement
