# Supplementary material for: Pancreatic Cancer Surveillance and Survival of High-Risk Individuals
Source: JAMA Oncol. 2024 Jul 3;10(8):1087–96. doi: 10.1001/jamaoncol.2024.1930 (PMC11223057; doi:10.1001/jamaoncol.2024.1930)
Supplement: Supplement 1. — eMethods eTable 1. Characteristics of 24 HRIs With PDAC With Known Tumor Location (Left) and Those Diagnosed During Screening (Right) Compared With Matched SEER Control Patients eTable 2. Comparison of 18 HRIs With Surgically Treated Surveillance-Detected PDAC and Matched SEER Control Patients With Surgically Treated PDAC eTable 3. Comparison of Overall PDAC Survival Among Subsets of HRIs With PDAC With Matched SEER Control Patients, With Additional Sensitivity Analysis Accounting for Potential Lead-Time Bias eFigure 1. Study Flow Chart eFigure 2. Overall Survival of 18 Surveillance-Detected HRIs With PDAC and Matched SEER Control Patients With Nonmetastatic, Resectable Disease [file jamaoncol-e241930-s001.pdf]

Supplemental Online Content

Blackford AL, Canto MI, Dbouk M, et al. Pancreatic cancer surveillance and survival of high-risk individuals. *JAMA Oncol*. Published online July 3, 2024.
doi:10.1001/jamaoncol.2024.1930

eMethods.

eTable 1. Characteristics of 24 HRIs With PDAC With Known Tumor Location (Left) and Those Diagnosed During Screening (Right) Compared With Matched SEER Control Patients

eTable 2. Comparison of 18 HRIs With Surgically Treated Surveillance-Detected PDAC and Matched SEER Control Patients With Surgically Treated PDAC

eTable 3. Comparison of Overall PDAC Survival Among Subsets of HRIs With PDAC With Matched SEER Control Patients, With Additional Sensitivity Analysis Accounting for Potential Lead-Time Bias

eFigure 1. Study Flow Chart

eFigure 2. Overall Survival of 18 Surveillance-Detected HRIs With PDAC and Matched SEER Control Patients With Nonmetastatic, Resectable Disease

This supplemental material has been provided by the authors to give readers additional information about their work.

© 2024 Blackford AL et al. *JAMA Oncology.*

eMethods

Study Subjects – SEER PDAC Control Matching

Twenty-six HRIs who were diagnosed with PDAC from 1998 to 2021 were matched to non-high-risk controls in the SEER Research Plus Data, 18 registries (Nov 2020 Submission). The HRIs cohort included N=26 patients who were diagnosed with PDAC. We re-staged one case who was diagnosed in 1998 using AJCC 6th edition criteria to be consistent with the SEER staging over time. From the SEER database, we extracted individuals diagnosed with PDAC at age 40 or older who were white race with histologic ICD-O-3 codes of C25.X (except for C25.4 which represents neuroendocrine tumors and were excluded) for cancer that was the individual's first primary cancer, flagged as having complete dates and follow-up for survival > 0 days with a known stage and not staged as T0. To ensure we extracted cases who were staged pathologically when surgery was performed and clinically staged when not, SEER controls who had surgery also had to have one or more nodes extracted, and cases who did not have surgery had to have zero nodes recorded as extracted.

AJCC staging definitions for PDAC have changed over the years, and the PDAC cases in the SEER database were staged according to the definitions in use at the time of diagnosis. To fairly compare stage at diagnosis between the HRIs and SEER cohorts, the HRI PDAC cases were also staged according to the staging definitions at the time of diagnosis. SEER controls with missing information on tumor size, stage, or node extraction were excluded. SEER controls diagnosed at autopsy were excluded.

We used the MatchIt package in R to select SEER PDAC controls who matched exactly on age, year of diagnosis and sex to HRI PDAC case. In this approach, all unique combinations of covariates (age, year of diagnosis, and sex) are used to form subclasses and only subclasses with both HRIs with PDAC and SEER PDAC controls are included in the final cohort. The 3 HRIs with PDAC diagnosed in 2020 and 2021 were matched to someone in SEER diagnosed in 2019; the one HRI PDAC case diagnosed in 1998 was matched to someone in SEER in 2004.

Sensitivity Analyses

We conducted three sets of sensitivity analyses for the comparison of stage and survival between HRIs with PDAC and matched SEER controls after sequentially excluding specific subgroups of HRIs with PDAC. First, the analyses described above were repeated after excluding two HRIs with PDAC with unknown primary tumor location that had developed PDAC several years after dropping out of surveillance and had presented with metastatic disease and treated at a local institution. The outcome of patients diagnosed with metastatic PDAC is considered to be driven by their metastatic disease and not their primary tumor location. The remaining N=24 HRIs with PDAC were re-matched to SEER controls while also including primary tumor location in the matching criteria (eTable 1, eTable 3).

© 2024 Blackford AL et al. *JAMA Oncology.*

Second, to assess the robustness of our results, we excluded four more HRIs with PDAC who had dropped out of their annual surveillance while limiting cases to those diagnosed within the screening program, leaving N = 20 in this analysis (eTable 3).

Third, the subgroup of N=18 HRIs with PDAC without metastatic disease who underwent surgery were re-matched to SEER controls using a coarsened exact matching approach, including age at diagnosis (by decade), year of diagnosis, sex, tumor location, and grade as matching criteria. We further restricted the SEER data to pancreatic cancer cases diagnosed with histologic ICD-O-3 codes C25.0 (head of pancreas) and C25.1 and C25.2 (body/tail of pancreas) only, AJCC staged 1 to 3, T stage 1 to 3, local or regional disease, tumor size greater than 0 cm and less than 6 cm, 1 or more nodes extracted, and had either distal or total pancreatectomy or Whipple surgery for their cancer. SEER controls with missing information on T, N, or M staging, grade, or the number of nodes extracted were excluded.

Results

Surgically Resectable Screen-Detected PDAC Cases

Eighteen of the 26 HRIs with PDAC were surveillance-detected and underwent surgical resection. These 18 HRIs with PDAC matched to the SEER controls according to age, sex, tumor location, tumor grade, and year of diagnosis were comparable with regards to type of surgical treatment (eTable 2).

The 18 HRIs with surveillance-detected resectable PDAC had a median OS of 144 months (12 years). Matched SEER controls had a median OS of 23 months. One and 5-year survival probabilities were 94% and 61% in the surgically-treated, screen-detected HRIs with PDAC and 74% and 24% in SEER controls, respectively (eFigure 1). These 18 HRIs with PDAC had a ~3-fold improved survival compared to their matched SEER controls. This difference was also not sensitive to the addition of a lead-time bias up to 12 months (eTable 3).

© 2024 Blackford AL et al. *JAMA Oncology.*

eTable 1. Characteristics of 24 HRIs With PDAC With Known Tumor Location (Left) and Those Diagnosed During Screening (Right) Compared With Matched SEER Control Patients

Characteristic	HRIs with PDAC with Known Primary Tumor Location	Matched SEER Controls	P value*	HRIs with PDAC Diagnosed During Surveillance	Matched SEER Controls	P value*
	N = 24	N = 510		N = 20	N = 414	
Age at diagnosis - mean (SD)	65.5 (9.7)	67.2 (8.3)		64.8 (9.5)	66.7 (7.8)	
Sex - no. (%)						
Female	14 (58.3)	294 (57.6)		13 (65.0)	265 (64.0)	
Male	10 (41.7)	216 (42.4)		7 (35.0)	149 (36.0)	
Year of diagnosis - no. (%)						
1998 – 2010	1 (4.2)	0 (0)		1 (5.0)	0 (0)	
2011 – 2015	2 (8.3)	39 (7.6)		1 (5.0)	10 (2.4)	
2016 – 2021	0 (0)	0 (0)		0 (0)	0 (0)	
Tumor Location - no. (%)						
Head	12 (50.0)	322 (63.1)		9 (45.0)	242 (58.5)	
Body or Tail	12 (50.0)	188 (36.9)		11 (55.0)	172 (41.5)	
Type of Surgery - no. (%)						
Whipple	9 (50.0)	93 (65)	0.93	9 (50.0)	68 (61.3)	0.93
Distal	8 (44.4)	39 (27.3)		8 (44.4)	35 (31.5)	
Total	1 (5.6)	11 (7.7)		1 (5.6)	8 (7.2)	
Unknown/No Surgery	6	367		2	303	
Tumor Size (cm) - median (range)	2.5 (0.6, 5)	3.5 (0.3, 8)	< 0.001	2.5 (0.6, 5)	3.5 (0.3, 8)	< 0.001
Grade - no. (%)						
1 (Well differentiated)	1 (5.3)	27 (15.4)	0.004	1 (5.3)	25 (18.5)	0.004
2 (Moderately differentiated)	15 (78.9)	84 (48)		15 (78.9)	65 (48.1)	
3 or 4 (Poorly differentiated/Undifferentiated/Anaplastic)	3 (15.8)	64 (36.6)		3 (15.8)	45 (33.3)	
Unknown Grade	5	335		1	279	
T stage - no. (%)						
I	6 (27.3)	16 (3.6)	0.001	6 (30.0)	13 (3.6)	< 0.001
II	10 (45.5)	159 (35.9)		8 (40.0)	135 (37.8)	
III	5 (22.7)	176 (39.7)		5 (25.0)	134 (37.5)	
IV	1 (4.5)	92 (20.8)		1 (5.0)	75 (21)	
Unknown	2	67		0	57	
N stage - no. (%)						
N0	12 (57.1)	266 (62.7)	0.71	12 (63.2)	216 (63.0)	0.53
N1	9 (42.9)	142 (33.5)		7 (36.8)	113 (32.9)	
N2	0 (0)	16 (3.8)		0 (0)	14 (4.1)	
Unknown	3	86		1	71	

© 2024 Blackford AL et al. *JAMA Oncology.*

Number of Positive Nodes/Total Nodes Examined - mean (SD)	0.06 (0.1)	0.16 (0.19)	0.02	0.06 (0.1)	0.16 (0.19)	0.02
M stage - no. (%)						
M0	19 (79.2)	254 (49.8)	< 0.001	19 (95)	198 (47.8)	< 0.001
M1	5 (20.8)	256 (50.2)		1 (5.0)	216 (52.2)	
AJCC stage - no. (%)						
1	10 (41.7)	58 (11.4)	< 0.001	10 (50.0)	47 (11.4)	< 0.001
2	8 (33.3)	134 (26.3)		8 (40.0)	101 (24.4)	
3	1 (4.2)	62 (12.2)		1 (5.0)	50 (12.1)	
4	5 (20.8)	256 (50.2)		1 (5.0)	216 (52.2)	
AJCC stage - no. (%)						
1a	6 (25.0)	13 (2.5)	< 0.001	6 (30.0)	10 (2.4)	< 0.001
1b	4 (16.7)	45 (8.8)		4 (20.0)	37 (8.9)	
2a	2 (8.3)	56 (11)		2 (10.0)	42 (10.1)	
2b	6 (25.0)	78 (15.3)		6 (30.0)	59 (14.3)	
3	1 (4.2)	62 (12.2)		1 (5.0)	50 (12.1)	
4	5 (20.8)	256 (50.2)		1 (5.0)	216 (52.2)	
AJCC stage - no. (%)						
Distant	5 (20.8)	256 (50.2)	< 0.001	1 (5.0)	216 (52.2)	0.002
Localized	12 (50.0)	114 (22.4)		12 (60.0)	89 (21.5)	
Regional	7 (29.2)	140 (27.5)		7 (35.0)	109 (26.3)	
Received neoadjuvant chemotherapy - no. (%)						
No/Unknown	21 (87.5)	169 (33.1)	< 0.001	17 (85.0)	126 (30.4)	< 0.001
Yes	3 (12.5)	341 (66.9)		3 (15.0)	288 (69.6)	

* P-values for differences between HRIs with PDAC and matched SEER controls, estimated from conditional logistic regression models. P values were not computed for age, sex, year of diagnosis, and tumor location, as they were the variables included in the matching algorithm.

** SEER data was available up through 2019. The HRIs with PDAC who was diagnosed in 2020 was matched to someone in SEER diagnosed in 2019

© 2024 Blackford AL et al. *JAMA Oncology.*

eTable 2. Comparison of 18 HRIs With Surgically Treated Surveillance-Detected PDAC and Matched SEER Control Patients With Surgically Treated PDAC

Characteristic	HRIs with PDAC N = 18	Matched SEER Controls N = 275	Eligible SEER Cohort N=9809	P value Compared to Matched SEER*
Age at diagnosis - mean (SD), years	64.5 (10.0)	67.3 (8.9)	65.7 (10.2)	NA
Sex - no. (%)				
Female	12 (66.7)	167 (60.7)	4741 (48.3)	NA
Male	6 (33.3)	108 (39.3)	5068 (51.7)	
Year of Diagnosis - no. (%)				
2004 – 2010	3 (33.3)	34 (12.5)	3781 (38.5)	NA
2011 – 2015	6 (33.3)	121 (44.0)	3938 (40.1)	
2016 – 2020 **	9 (50.0)	120 (43.6)	2090 (21.3)	
Tumor Location – no. (%)				
Head	9 (50)	217 (78.9)	7730 (78.8)	NA
Body or Tail	9 (50)	58 (21.1)	2079 (21.2)	
Type of Surgery – no. (%)				
Whipple	9 (50)	177 (64.4)	6211 (63.3)	0.90
Distal	8 (44.4)	75 (27.3)	2560 (26.1)	
Total Pancreatectomy	1 (5.6)	23 (8.4)	1038 (10.6)	
Tumor Size, cm - median (range)	2.5 (0.6, 5)	3.1 (0.1, 6)	3 (0.1, 6)	< 0.001
Grade – no. (%)				
1 (Well-Differentiated)	1 (5.6)	1 (0.4)	1841 (18.8)	
2 (Moderately Differentiated)	14 (77.8)	270 (98.2)	4637 (47.3)	
3 or 4 (Poorly Differentiated, Undifferentiated, or Anaplastic)	3 (16.7)	4 (1.5)	3331 (34)	
T Stage – no. (%)				
I	6 (33.3)	9 (3.3)	880 (9)	<0.001
II	8 (44.4)	58 (21.1)	1664 (17)	
III	4 (22.2)	208 (75.6)	7265 (74.1)	
N Stage – no. (%)				
N0	12 (66.7)	87 (31.6)	3639 (37.1)	0.10
N1	6 (33.3)	172 (62.5)	6075 (61.9)	
N2	0 (0)	16 (5.8)	95 (1)	
AJCC Stage – no. (%)				
1	10 (55.6)	31 (11.3)	1604 (16.4)	< 0.001
2	8 (44.4)	226 (82.2)	8088 (82.5)	
3	0 (0)	18 (6.5)	117 (1.2)	
AJCC Stage – no. (%)				
Localized	12 (66.7)	87 (31.6)	3639 (37.1)	0.02
Regional	6 (33.3)	188 (68.4)	6170 (62.9)	
Number of Positive Nodes/Total Nodes Examined – mean (SD)	0.06 (0.10)	0.17 (0.2)	0.15 (0.19)	0.02
Received Chemotherapy – no. (%)				
Yes	3 (16.7)	196 (71.3)	6272 (63.9)	< 0.001
No/Unknown	15 (83.3)	79 (28.7)	3537 (36.1)	

* P-values differences between HRIs with PDAC and matched SEER controls, estimated from conditional logistic regression models. P values were not computed for age, sex, year of diagnosis, tumor location, and tumor grade as they were the variables included in the matching algorithm.
** SEER data was available up through 2019. The HRIs with PDAC who was diagnosed in 2020 was matched to someone in SEER diagnosed in 2019

© 2024 Blackford AL et al. *JAMA Oncology.*

eTable 3. Comparison of Overall PDAC Survival Among Subsets of HRIs With PDAC With Matched SEER Control Patients, With Additional Sensitivity Analysis Accounting for Potential Lead-Time Bias

	N	Median OS (months)	Survival Probability (%)		Hazard Ratio 95% CI, P*			
			1 Year	5 year	Observed Data	3-month Lead Time Bias	6-month Lead Time Bias	12-month Lead Time Bias
Limited to HRIs with PDAC with Known Tumor Location								
HRIs with PDAC	24	61.7	91 [80, 100]	55 [35, 86]	1.0 (reference)			
Matched SEER Controls	510	9	38 [34, 43]	8 [5, 13]	5.03 [2.57, 9.87] P<0.001	4.73 [2.37, 9.45] P<0.001	4.49 [2.22, 9.08] P<0.001	4.04 [1.97, 8.29] P<0.001
Limited to HRIs with PDAC Who Were Diagnosed in Screening								
HRIs with PDAC	20	144	95 [86, 100]	61 [40, 93]	1.0 (reference)			
Matched SEER Controls	414	9	41 [36, 46]	9 [5, 14]	5.88 [2.72, 12.67] P<0.001	5.59 [2.54, 12.29] P<0.001	5.38 [2.43, 11.91] P<0.001	4.82 [2.14, 10.86] P<0.001
Limited To HRIs with PDAC Diagnosed with Resectable Disease								
HRIs with PDAC	18	144	94 [84, 100]	61 [39, 97]	1.0 (reference)			
Matched SEER Controls	275	23	74 [69, 80]	24 [18, 31]	3.30 [1.62, 6.73] P=0.001	3.15 [1.56, 6.35] P=0.001	2.98 [1.48, 5.98] P=0.002	2.66 [1.28, 5.51] P=0.008

* Hazard ratios from Cox proportional hazards models estimated with cluster-robust standard errors, clustered on the matched subclass and including subclass-specific weights. Analyses in the top two panels (cases with known tumor location and cases diagnosed in the screening program) reflect data matched to SEER controls on age, sex, year of diagnosis, and tumor location. Analyses among HRIs with PDAC diagnosed with resectable disease reflect data matched to SEER controls on age, sex, year of diagnosis, tumor location, and tumor grade.

© 2024 Blackford AL et al. *JAMA Oncology.*

eFigure 1. Study Flow Chart

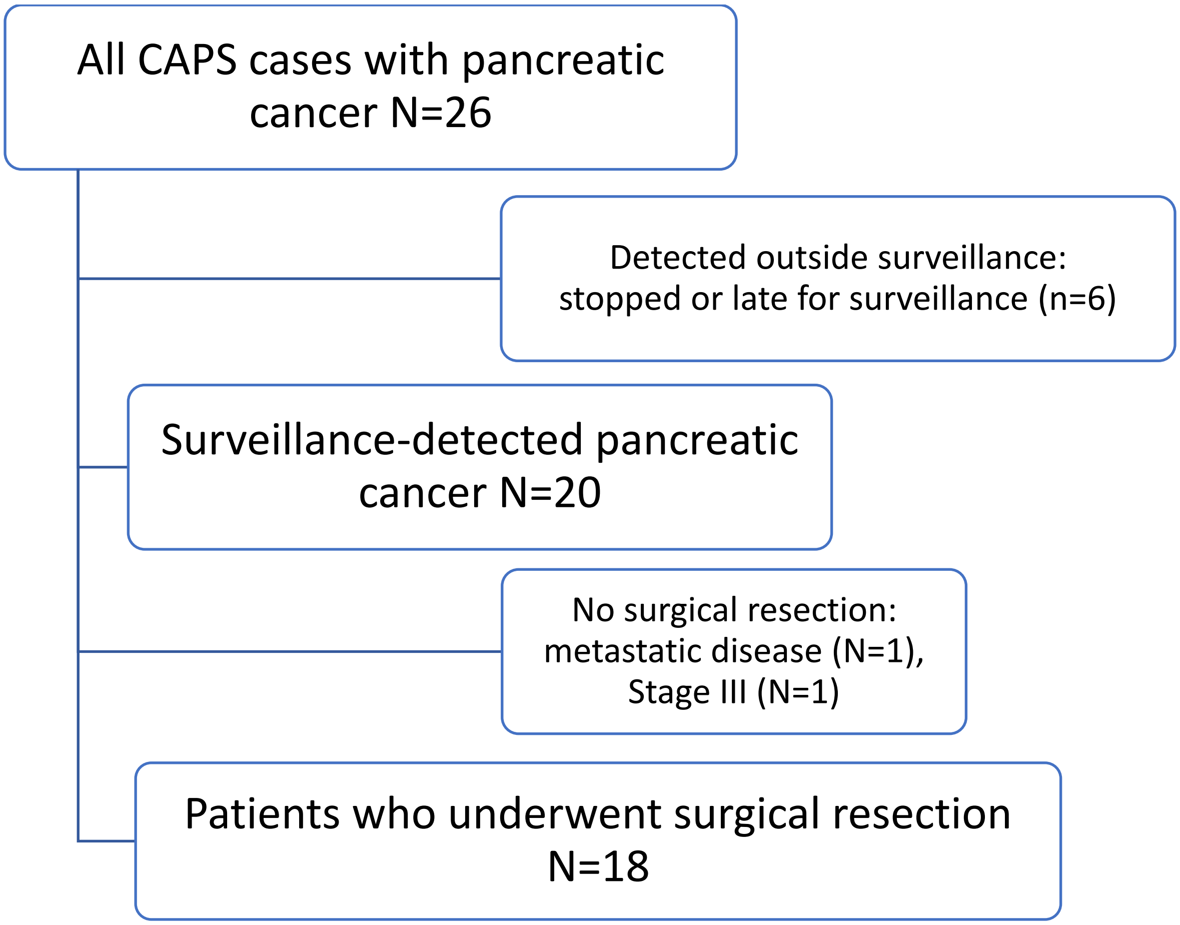

© 2024 Blackford AL et al. *JAMA Oncology.*

eFigure 2. Overall Survival of 18 Surveillance-Detected HRIs With PDAC and Matched SEER Control Patients With Nonmetastatic, Resectable Disease

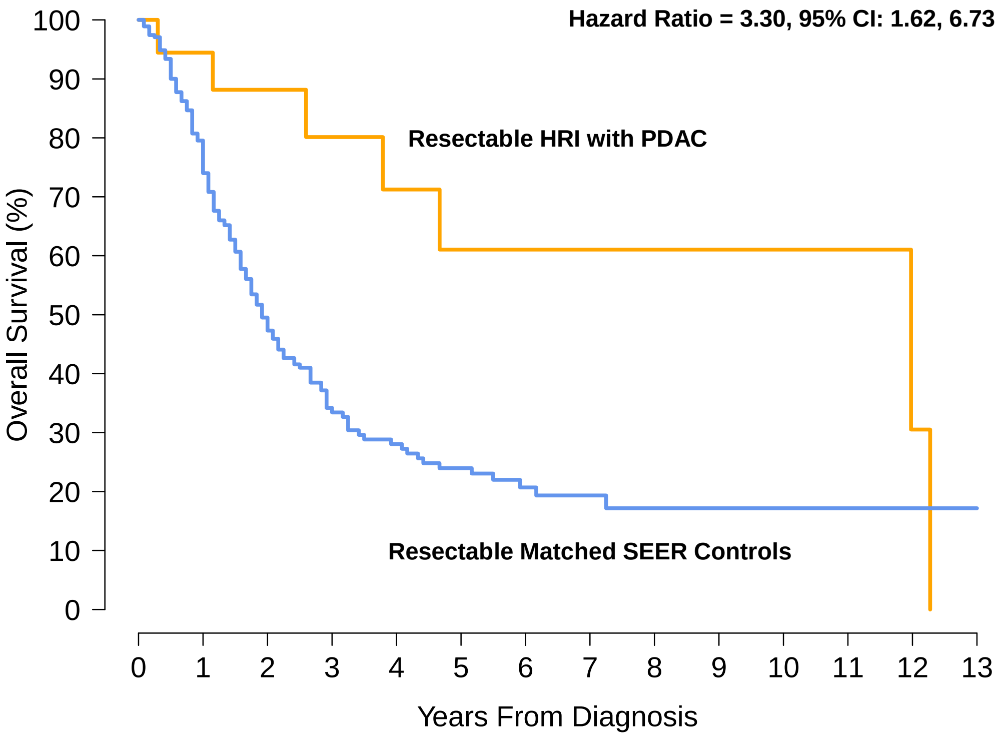

No. at risk														
Resectable HRI with PDAC	18	15	13	10	8	6	5	5	2	2	2	2	1	0
Resectable Matched SEER Controls	275	201	112	45	36	29	16	9	7	7	3	2	2	2

© 2024 Blackford AL et al. *JAMA Oncology.*
