# Supplementary material for: Pancreatic Cancer Surveillance and Survival of High-Risk Individuals
Source: JAMA Oncol. 2024 Jul 3;10(8):1087–96. doi: 10.1001/jamaoncol.2024.1930 (PMC11223057; doi:10.1001/jamaoncol.2024.1930)
Supplement: Supplement 2. — Data Sharing Statement [file jamaoncol-e241930-s002.pdf]

Data Sharing Statement

Blackford. Pancreatic Cancer Surveillance and Survival of High-Risk Individuals. *JAMA Oncol*. Published July 03, 2024. doi:10.1001/jamaoncol.2024.1930

Data
Data available: No

Additional Information
Explanation for why data not available: The data collected was from studies fully supported by multiple sponsors, including the National Insitutes of Health, Lustgarten Foundation, other private organizations.
